# Source Apportionment and Ecological Risk Assessment of Heavy Metals in Urban Fringe Areas: A Case Study of Kaifeng West Lake, China

**DOI:** 10.3390/toxics13090720

**Published:** 2025-08-27

**Authors:** Jinting Huang, Bingyan Jin, Feng Zhou

**Affiliations:** 1 College of Surveying and Mapping Engineering, Yellow River Conservancy Technical University, Kaifeng 475004, China; 2 Faculty of Geographical Science, Beijing Normal University, Beijing 100875, China; 202221490026@mail.bnu.edu.cn; 3 Hubei Institute of Surveying and Mapping, Wuhan 430074, China; hbchzf@sohu.com

**Keywords:** heavy metal, ecological risk, sources identification, lake sediments, urban fringe areas

## Abstract

Exploring the pollution characteristics and ecological risks of urbanization on lakes in urban fringe areas has guiding significance for the control and scientific management of heavy metal pollution in lakes in urban fringe areas. Taking the West Lake in Kaifeng city as an example, the samples of the sediments and surface water of the lake were collected, and the contents of heavy metals (As, Cd, Cr, Cu, Ni, Pb, and Zn) were measured, assessing the degree and ecological risk of heavy metal pollution using the Geo-Accumulation Index (*I_geo_*) and Potential Ecological Risk Index methods (RI); and the sources of pollution were identified. The results show that the heavy metal concentrations in the surface water of the West Lake in Kaifeng city are generally low; average concentrations of Cd, Cu, Zn, Cr, Ni, Pb, and As in sediments are 3.120, 1.810, 1.700, 1.540, 1.000, 0.990, and 0.430 times higher than the background value of fluvo-aquic soil, respectively. The sequence of the average *I_geo_* from high to low is Cd (1.020) > Cu (0.220) > Zn (0.160) > Cr (0.000) > Pb (−0.610) > Ni (−0.640) > As (−1.850). Among them, contaminations with Pb are classed as moderately polluted; As pollution is relatively light, while other heavy metals are unpolluted. The average Potential Ecological Risk Coefficient (*E*) values for seven heavy metals are Cd (93.500) > Cu (9.040) > Ni (4.990) > Pb (4.950) > As (4.290) > Cr (3.080) > Zn (1.700). Cd is at a considerable potential ecological risk, while other heavy metals are at low ecological risks. Heavy metal pollution in sediment of West Lake in Kaifeng mainly comes from traffic activities such as yacht machinery wear and gasoline burning. The research findings provide a scientific foundation for developing effective mitigation strategies against heavy metal contamination in peri-urban lacustrine ecosystems.

## 1. Introduction

Urban lacustrine water bodies constitute integral components of metropolitan wetland ecosystems, playing critical roles in maintaining regional hydrological cycles and modulating urban climate regimes [1]. However, rapid economic development in recent decades has led to increasingly severe lake pollution. Heavy metals are a typical pollutant in lake pollution, characterized by their persistence, strong biological toxicity, and magnification in the food chain [2,3,4]. They can pose long-term and persistent potential risks to human health and ecological safety [5]. Sediments serve as both the source and sink for heavy metal pollution in lake water [6,7], significantly impacting the quality of lake ecosystems [7,8]. Therefore, investigating the contamination characteristics, ecological risks, and source apportionment of heavy metals in urban lake sediments holds significant importance for addressing ecological degradation and promoting sustainable urban development.

The urban fringe is a dynamic, unified, and transitional spatial entity situated between urban and rural areas, influenced by both urban and rural factors, and characterized by natural and social attributes. Existing studies have primarily focused on examining the social dimensions of this distinct region, such as land use change, spatial structure evolution, functional characteristics, and developmental impacts [9,10]. However, with the advancement of urbanization, the ecological environmental issues in the urban fringe are increasingly prominent, and the academic focus is shifting towards the ecological environment aspect [11,12]. This is critical for addressing ecological degradation in peri-urban areas and advancing sustainable urban development. Heavy metal pollution in lake sediments has always been one of the key ecological environmental issues that academia focuses on [13]. In recent years, with the accelerated process of urbanization, research on heavy metal pollution in urban lake sediments has gradually deepened, and the findings have indicated varying degrees of ecological risks [14,15]. While urban lake pollution is well-studied, transitional ecosystems at urban–rural fringes remain overlooked, particularly regarding the apportionment of heavy metal sources in sediments. Few studies have also examined heavy metal contamination in sediments from lakes in Kaifeng’s peri-urban areas. As critical pollutants threatening aquatic ecosystems, heavy metals require further investigation into their spatial distribution, source allocation, and ecological risks in lake sediments within urban fringe areas to uncover their linkages with rapid urbanization.

Kaifeng is renowned as the “Water City of Northern China”, with multiple water bodies within the city area. West Lake, located in the western part of Kaifeng’s new district, is a typical urban fringe lake. West Lake serves various functions, such as supporting agricultural irrigation, providing industrial and domestic water supply to the urban area, and improving the ecological environment. However, with the rapid acceleration of urbanization and the continuous intensification of agricultural intensification, the accumulation of heavy metals in West Lake in Kaifeng is also worsening. Therefore, leveraging the dual characteristics of peri-urban areas at the urban–rural interface, this study systematically integrates lake sediment pollution with their distinct spatial setting. The pollution levels and ecological risks of West Lake are evaluated using the Geo-Accumulation Index method and an improved Potential Ecological Risk Index method. Additionally, spatial variations in sediment pollution are analyzed, and the sources of heavy metals in sediment are investigated using methods such as Pearson correlation analysis, principal component analysis, cluster analysis, and positive matrix factorization (PMF). This study aims to establish a theoretical foundation for controlling pollution sources and implementing targeted remediation of high-risk heavy metals in Kaifeng West Lake and similar peri-urban lake ecosystems.

## 2. Materials and Methods

### 2.1. Study Area

Kaifeng City is located in the eastern part of the plain region of the middle and lower reaches of the Yellow River. It has a temperate continental monsoon climate, with an average annual temperature of 14 °C and an average annual precipitation of 627.5 mm. The parent material in Kaifeng City is predominantly alluvial deposits from the Yellow River, and the primary soil type is predominantly fluvial soil [3,4]. West Lake in Kaifeng was established in 2010, and water storage began in May 2014. It is supplied by the Xigan Canal, which diverts water from the Yellow River. The reservoir covers an area of 1.984 km^2^, with an average depth of 2.5 m and a total capacity of 8.34 million m^3^. The reservoir area is part of the alluvial fan of the Yellow River, characterized by lower elevation in the southeast and higher elevation in the northwest. The terrain within the area is flat and open. West Lake serves as a water storage reservoir for the Yellow River irrigation area. It is located in the western part of Kaifeng’s new district, connecting the old and new urban areas, and functions as an urban ecological scenic area that combines water storage, flood control, irrigation, and tourism.

### 2.2. Sample Collection and Analysis

**Figure 1:**
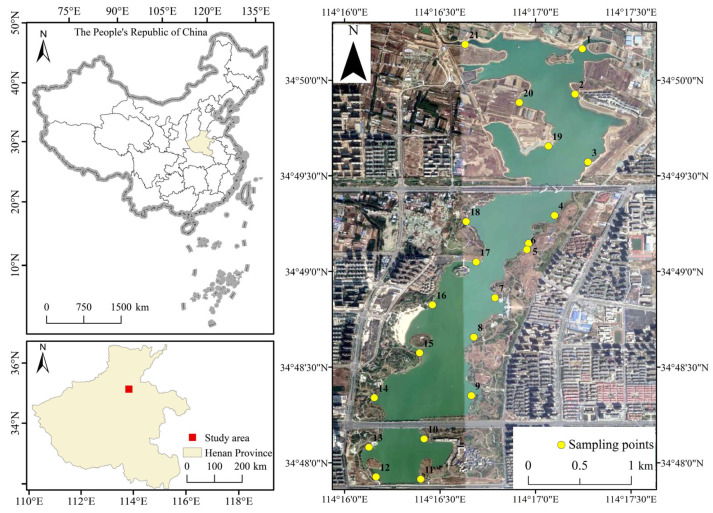
Distribution of sampling points in the study area.

The samples were collected in September 2020. Due to the elongated nature of the study area, sampling points were evenly distributed around the lake, avoiding pollution sources and stagnant water areas to ensure that the sampling points represented the overall condition of the lake (Figure 1). A dredging-type sediment sampler was used to collect 21 composite samples of lake bottom sediment from depths ranging from 0 to 0.2 m. Approximately 500 mL water samples were collected at a depth of 0.5 m below the water surface using a water sampler. A GPS locator was used to record the coordinates of all sampling points. After collection, sediment samples were air-dried in a controlled laboratory environment (temperature: 20–25 °C; relative humidity: 40–60%) with periodic manual turning until constant weight was achieved (mass variation < 0.1% over 24 h, confirmed by analytical weighing). The dried samples were then crushed, ground, and sieved through a 100-mesh (0.149 mm) nylon screen to remove impurities. The samples were then digested with a HNO_3_-HF-HClO_4_ mixture for subsequent heavy metal concentration determination. Water samples were transported back to the laboratory and filtered through a 0.45 μm microfiltration membrane. The filtered samples were stored in polyethylene bottles at 0–4 °C after acidification for analysis.

All pretreated samples were first subjected to preliminary quantification of heavy metal concentrations using an AA-6601F atomic absorption spectrophotometer (Shimadzu, Tokyo, Japan) to establish analytical ranges. The concentrations of Cd, Cr, Cu, Ni, Pb, and Zn were determined using an inductively coupled plasma mass spectrometer (ICP-MS; Thermo Fisher Scientific, Waltham, MA, USA) with detection limits of 0.1 μg·L^−1^ for Cr and Ni, and 0.01 μg·L^−1^ for Cd, Cu, Pb, and Zn. The concentrations of As were determined using atomic fluorescence spectrometry (AFS; AFS-3100, Skyray Instrument, Dallas, TX, USA) after sample digestion with HNO_3_:HCl (1:3), with a detection limit of 0.01 μg·L^−1^ for As. National standard soil samples (GSS-2), duplicate samples, and blank samples were included during the analysis to ensure accuracy and precision. The relative deviations of the duplicate samples were all less than 5%, and the recoveries of the spiked elements ranged from 94.62% to 109.74%, indicating that the analysis results met the quality control requirements.

### 2.3. Research Methods

#### 2.3.1. Geo-Accumulation Index Method

The Geo-Accumulation Index (*I_geo_*) is a quantitative indicator proposed by German scholar Müller [16] in 1969 for studying the level of heavy metal pollution in sediments. It considers anthropogenic factors, geochemical background values, and the influence of natural diagenesis on background value variations. Therefore, it is widely used for the assessment of heavy metal pollution in river and lake sediments. The calculation expression is as follows:

(1) $$I_{geo}=\log_{2}[C_{i}/(k\times B_{i})]$$

In the equation, *I_geo_* represents the Geo-Accumulation Index, *C_i_* represents the concentration of heavy metal element *i* in the sediment, and *B_i_* represents the environmental geochemical background value of that heavy metal element. For this study, the Chinese tidal soil element background values were selected [17]. The parameter *k* is used to account for potential variations in background values due to geological differences and is commonly set to 1.5. According to *I_geo_*, the level of heavy metal pollution in sediments is categorized into seven levels: *I_geo_* ≤ 0, no pollution; 0 < *I_geo_* ≤ 1, slight pollution; 1 < *I_geo_* ≤ 2, moderate pollution; 2 < *I_geo_* ≤ 3, moderate to strong pollution; 3 < *I_geo_* ≤ 4, strong pollution; 4 < *I_geo_* ≤ 5, strong to extremely strong pollution; and *I_geo_* > 5, extremely strong pollution.

#### 2.3.2. Potential Ecological Risk Index Method

The Potential Ecological Risk Index method is an approach proposed by Swedish geochemist Hakanson [18] in 1980 for assessing the risk of heavy metal pollution in aquatic sediments. This method is based on sedimentology and evaluates the contamination of heavy metals in sediments based on their properties and environmental behaviors. It considers not only the concentration of each heavy metal but also the toxicity level, environmental effects, and overall pollution level of the heavy metals. The calculation formula is as follows:

(2) $$E_{r}^{i}=T_{r}^{i}\times \frac{C_{s}^{i}}{C_{n}^{i}}$$

(3) $$\mathrm{RI}=\sum_{i=1}^{n}E_{r}^{i}=\sum_{i=1}^{n}T_{r}^{i}\times C_{f}^{i}=\sum_{i=1}^{n}T_{r}^{i}\times \frac{C_{s}^{i}}{c_{n}^{i}}$$

In the equation, $E_{r}^{i}\;$ represents the potential ecological risk factor for the heavy metal *i*; $C_{s}^{i}\;$ is the measured concentration of the heavy metal *i*; $C_{n}^{i}$ is the reference value for evaluating the heavy metal *i*; in this study, the Chinese coastal soil element background value [17] is used as the reference value; $T_{r}^{i}\;$ represents the toxicity response coefficient for the heavy metal *i*. RI is the comprehensive Potential Ecological Risk Index for heavy metals. *T_r_* values for Cd, As, Cu, Pb, Ni, Cr, and Zn are 30, 10, 5, 5, 5, 2, and 1, respectively.

The individual Potential Ecological Risk Coefficient (*E*) and the comprehensive Potential Ecological Risk Index (RI) classification criteria must be adjusted based on the specific types and quantities of pollutants being evaluated. However, many existing studies have directly adopted Hakanson’s classification criteria, which can affect the reliability of the assessment results [19]. In this study, based on relevant research [4,19] and the actual evaluation of heavy metals, the Potential Ecological Risk Index (Table 1) has been adjusted. The upper limit values for the risk levels in the *E* value classification are determined by multiplying the non-polluting pollution coefficient (*C* = 1) along with the maximum toxicity coefficient of the evaluated pollutants. The upper limit values for the other risk levels are obtained by multiplying the classification value of the previous level by 2. In this study, among the seven heavy metals, Cd has the highest *T* value (30), which serves as the first level threshold, based on which the classification criteria for *E* in this study are derived. For adjusting the classification criteria for RI, firstly, the first level threshold value (150) according to Hakanson is divided by the sum of toxicity coefficients for the 8 pollutants (133) to obtain the unit toxicity coefficient value for RI (1.13); then, the unit toxicity coefficient value for RI is multiplied by the sum of toxicity coefficients for the seven heavy metals in this study (58), the tens digit is taken to obtain the first level threshold value for RI (1.13 × 58 = 65.54 ≈ 70), and the classification values for other levels are obtained by multiplying the classification value of the previous level by 2.

#### 2.3.3. Positive Matrix Factorization (PMF) Method

In this study, the quantification and source apportionment of heavy metals in sediment were conducted using the PMF 5.0 model developed by the United States Environmental Protection Agency (EPA) [20,21]. PMF is a mathematical method based on receptor modeling that utilizes sample composition to quantitatively analyze pollution sources. PMF addresses a critical limitation of conventional receptor models: it quantifies source contributions while preserving non-negativity constraints (source profiles and contributions ≥ 0), which aligns with environmental reality. It employs a least squares iterative algorithm to decompose the receptor’s original data matrix (*X*) into source contribution matrix (*G*), source profile matrix (*F*), and residual matrix (*E*). The source contribution matrix (*G*) has rows corresponding to sampling locations and columns representing pollution sources, quantifying the contribution share of each source. The source profile matrix (*F*) contains rows defining pollution sources and columns indicating chemical species, characterizing source compositions. The residual matrix (*E*) encapsulates the uncertainty inherent in the model. PMF has been widely applied for pollutant source apportionment in the atmosphere [22,23], water [24,25], and sediment [26]. Its formula is as follows:

(4) $$X_{ij}=\sum_{k=1}^{p}G_{ik}F_{jk}+E_{ij}\;$$

In the formula, *X_ij_* represents the concentration of element *i* in the *j*-th sample, *G_ik_* represents the concentration of element *i* in source *k*, *F_jk_* represents the contribution value of source *k* to element *i*, *E_ij_* represents the residual matrix, and *p* represents the number of factors.

(5) $$Q=\sum_{i=1}^{m}\sum_{j=1}^{n}{\left(\frac{E_{ij}}{u_{ij}}\right)}^{2}$$

In the formula, *Q* represents the objective function, and *u_ij_* represents the uncertainty of element *i* in the *j*-th sample.

(6) uij=56×MDL,Xij≤MDL

(7) $$u_{ij}=\sqrt{{\left(\sigma \times X_{ij}\right)}^{2}+{\left(0.5\times MDL\right)}^{2}},\;X_{ij}>MDL$$

In the formula, *σ* represents the error fraction, and *MDL* represents the method detection limit.

## 3. Results

### 3.1. Analysis of Heavy Metal Content in Surface Water and Sediment

Analysis of surface water from Kaifeng West Lake (Table 2) revealed that the concentrations of six monitored heavy metals, except for Ni, were below the Class IV concentration limits stipulated in the “Environmental Quality Standards for Surface Water” (GB 3838-2002) [27]. Ni concentrations, while detectable, also fell below the specific project standard limits for centralized domestic drinking water surface sources (GB 3838-2002). Collectively, the surface water analysis indicates a clean state for the lake.

Heavy metals and other pollutants tend to accumulate in lake sediments, and the quality of the water body cannot be accurately assessed solely based on the concentration of pollutants in surface water. As shown in Table 3, the average contents of Cd, Cu, Zn, Cr, Ni, Pb, and As in sediment are 3.120, 1.810, 1.700, 1.540, 1.000, 0.990, and 0.430 times higher than the background values in tidal soil [17], respectively. Among them, Cd pollution is the most severe, while Ni, Pb, and As do not exceed the background values. The coefficient of variation for the seven heavy metals in the sediment of Kaifeng West Lake ranges from 14.37% to 28.48%, with Cu having the highest coefficient of variation and showing the most significant spatial variation.

### 3.2. Spatial Distribution of Heavy Metal Pollution in Sediment

**Figure 2:**
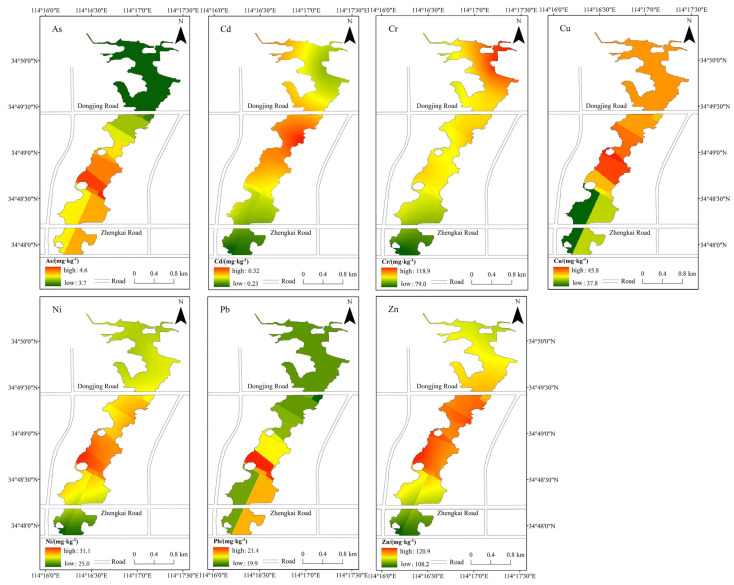
Spatial distribution of heavy metal concentration in the sediment of Kaifeng West Lake.

Based on the fitting of the variogram function, ordinary kriging interpolation was conducted to determine the spatial distribution of heavy metal concentrations in sediment within the study area. The spatial distribution of the seven heavy metals is shown in Figure 2. High concentrations of Cd, Cu, and Cr were predominantly clustered in the central and northern lake areas, with Cd and Cu peaking south of East Road and Cr north of Dongjing Road. Elevated Pb and As levels were mainly found in the central and southern regions, peaking centrally. Ni and Zn pollution were primarily concentrated in the central lake. Critically, a common spatial pattern emerged: the highest concentrations for all metals consistently occurred in the central lake, specifically between Dongjing Road and Zhengkai Road. This distribution correlates strongly with the location of artificial beaches and recreational facilities (e.g., yachts) along the western shore of the central lake area.

### 3.3. Sediment Heavy Metal Pollution and Risk Assessment

#### 3.3.1. Evaluation Results Using the Geo-Accumulation Index Method for Sediment Heavy Metal Pollution and Risk Assessment

The calculation results of the Geo-Accumulation Index for the seven heavy metals in the sediments of Kaifeng West Lake (Table 4), indicate the average *I_geo_* values for the seven heavy metals in the following order: Cd (1.020) > Cu (0.220) > Zn (0.160) > Cr (0.000) > Pb (−0.610) > Ni (−0.640) > As (−1.850). Cd shows a moderate (Level 2) pollution level and has the highest degree of pollution, with 57.1% of the sampling points showing moderate (Level 2) pollution. Zn and Cu show mild (Level 1) pollution. Cr, Pb, Ni, and As are generally in a relatively clean state. Overall, Cd exhibited the highest contamination levels among the heavy metals analyzed.

#### 3.3.2. Evaluation Results of the Potential Ecological Risk Index Method

**Figure 3:**
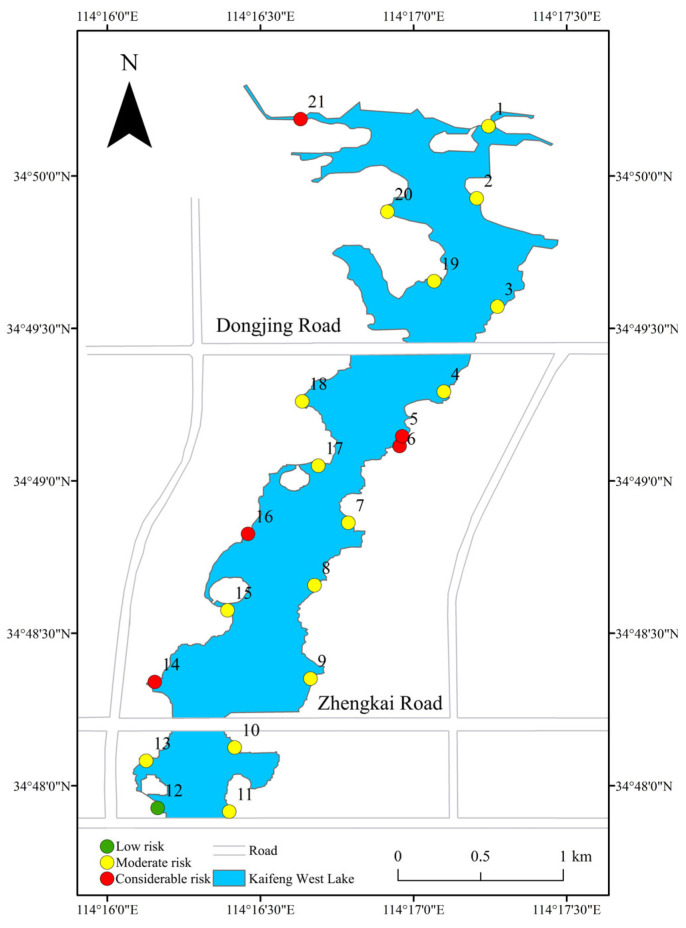
Spatial distribution of the potential ecological risk index for heavy metals in the sediment of Kaifeng West Lake.

Referring to the background values of elemental content in Chinese tidal soil [17], the Potential Ecological Risk Coefficient (*E*) for individual heavy metals and the comprehensive Potential Ecological Risk Index (RI) were calculated. Based on the ecological risk indices for individual heavy metals, the average *E* values for the eight heavy metals in Kaifeng West Lake are as follows in descending order: Cd (93.500) > Cu (9.040) > Ni (4.990) > Pb (4.950) > As (4.290) > Cr (3.080) > Zn (1.700). For Cd, sample point 12 was at a moderate ecological risk level, while sample points 5 and 6 were at high ecological risk levels. The remaining sample points were at moderate ecological risk levels, resulting in an overall moderate ecological risk classification for Cd. For Cr, Cu, Ni, Pb, Zn, and As, all sampling points were at slight ecological risk levels. Spatially, the RI distribution (Figure 3) revealed that in the eastern region, all sample points exhibited moderate ecological risk levels except sample points 5 and 6, which were at high ecological risk levels. In the western region, sample point 12 showed slight ecological risk, while sample points 14, 16, and 21 were at high ecological risk levels. The remaining western sample points were at moderate ecological risk levels. Collectively, Kaifeng West Lake was assessed to be at a moderate ecological risk level.

### 3.4. Source Analysis of Heavy Metals

#### 3.4.1. Pearson Correlation Analysis Results

Pearson correlation analysis can be used to elucidate the correlation between heavy metals in sediment and investigate the direction and degree of their correlation. The statistical significance of Pearson correlation is determined through T-tests before conducting the analysis. If there is a significant correlation between each pair of heavy metals, it indicates that they may have similar pollution sources [28]. Pearson correlation analysis was performed on seven heavy metal elements in the sediment of Kaifeng West Lake using SPSS 22.0 software. The results are shown in Table 5. Cd shows a significant positive correlation with Cu, Ni, Pb, Zn, and As. Cr only exhibits a significant positive correlation with Ni. In addition to a significant positive correlation with Cd, Cu also shows significant positive correlations with Ni and Zn. Ni demonstrates a strong positive correlation with all other heavy metals, with correlation coefficients exceeding 0.62. Pb exhibits strong, significant correlations with Cd, Ni, Zn, and As, among others. Zn displays strong, significant correlations with Cd, Cu, Ni, Pb, and As. As shows significant correlations with Cd, Ni, Pb, and Zn.

#### 3.4.2. Principal Component Analysis Results

**Figure 4:**
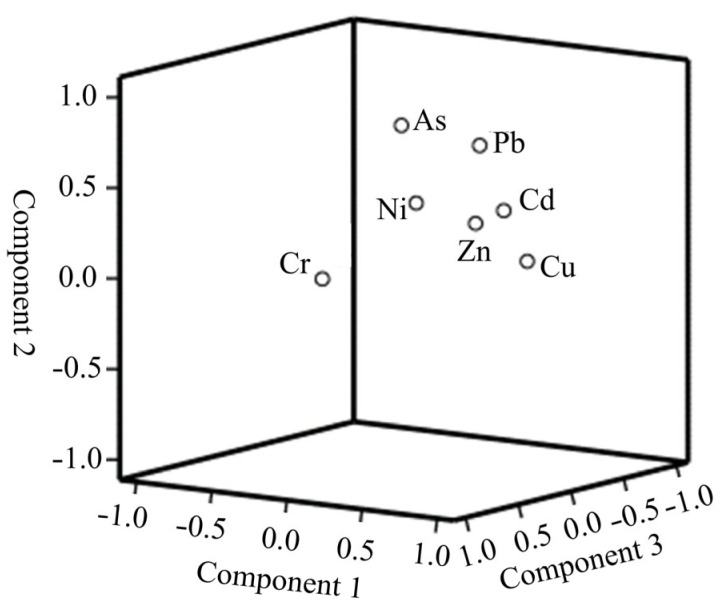
Loadings plot of principal component analysis.

In this study, principal component analysis was conducted on the heavy metals in the sediment to analyze their sources. The suitability of the experimental data was verified using the Kaiser–Meyer–Olkin and Bartlett’s sphere tests [29]. In this study, the KMO value was found to be 0.78, and the probability value of the Bartlett’s sphere test was less than 0.01, indicating that the results of the principal component analysis are reliable [30]. The seven heavy metals were classified into three groups (Figure 4): the first principal component is mainly composed of Cd, Cu, and Zn, explaining 35.99% of the variance; the second principal component is mainly composed of Pb and As, explaining 27.15% of the variance; the third principal component is mainly composed of Cr and Ni, explaining 22.45% of the variance.

#### 3.4.3. Cluster Analysis Results

**Figure 5:**
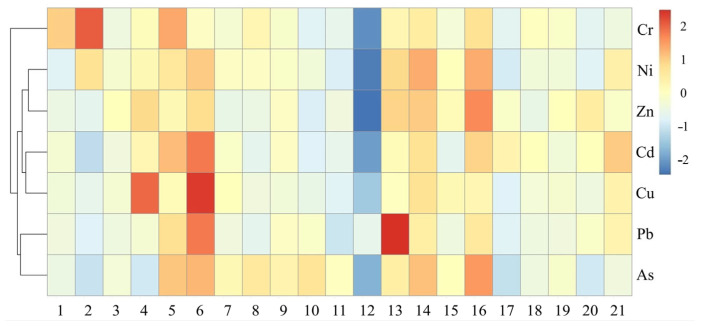
Heatmap of cluster analysis for heavy metals in sediments of Kaifeng West Lake.

Cluster analysis was performed using a clustering heatmap to further analyze the sources of heavy metals in the sediment. After standardizing the data, Ward.D method was used for systematic clustering analysis of the seven heavy metals in Kaifeng West Lake sediment based on Euclidean distance. The seven heavy metals can roughly be classified into three categories (Figure 5): the first category consists of Cr; the second category includes Cd, Cu, Ni, and Zn; the third category comprises Pb and As. The results of cluster analysis are generally consistent with those of principal component analysis.

#### 3.4.4. PMF Analysis Results

**Figure 6:**
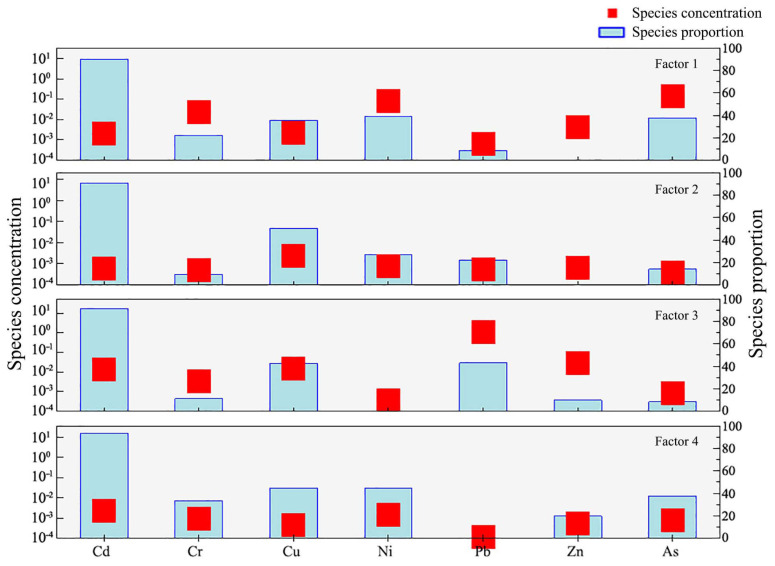
Contribution rates of heavy metal elements in each factor.

In this study, the PMF model was further employed for quantitative source apportionment of heavy metals in sediment. The signal-to-noise ratio (S/N) for all elements in the model was greater than 2, indicating good data quality. The initial setting for the number of factors in the base model was 2 to 4, with 20 iterations. Ultimately, after evaluation, the optimal number of factors was determined to be 4, resulting in the lowest and most stable *Q* values, with residuals of all elements falling within the range of −3 to 3. The model’s predictions show good correlation with the measured values (R^2^ > 0.6), with Cd, Cu, and Pb exhibiting R^2^ values higher than 0.99. The results of the model run (Figure 6) indicate that Factor 1 has the highest contribution rate (34.9%), with As (57.0%), Ni (52.7%), and Cr (42.8%) being the major loadings. Factor 3 has the second-highest contribution rate (34.3%), with significant loadings of Pb (70.8%), Zn (42.8%), Cu (38.0%), and Cd (37.1%). Factor 2 has a contribution rate of 15.8% and is primarily loaded with Cu. Factor 4 has the smallest contribution rate (15.0%) and is mainly loaded with Cd (24.7%) and Ni (21.2%).

## 4. Discussion

Comparisons were made between the Baogong Lake, Longting Lake, and Tietat Lake in the urban area of Kaifeng City [31] and the Baiyun Lake in Guangzhou City [32], Longyang Lake and Moshui Lake in Wuhan City [33], as well as the distant lakes of Hulun Lake [34], Qinghai Lake [35], and Chenghai Lake [36]. The results are shown in Table 6. Baogong Lake, Longting Lake, and Tietat Lake are the three main water bodies in the urban area of Kaifeng City. Except for the Cr element, the average levels of other heavy metal elements in the sediment of Baogong Lake are generally higher than those in West Lake in Kaifeng, particularly for Pb and Cu, which are 4.02 and 2.84 times higher, respectively. This could be attributed to the crossing of the main road, Yingbin Road, through Baogong Lake in the city area. In comparison, the heavy metal content in Longting Lake and Tietalu Lake is mostly lower than that in Kaifeng West Lake, which may be related to the fact that both lakes are located within scenic areas. Longting Lake and Tieta Lake are respectively situated in Longting Park and Tieta Park, which are mainly frequented by pedestrians and do not allow vehicle traffic. The heavy metal content in the sediment of Baiyun Lake in Guangzhou is much higher than that in Kaifeng West Lake (1.43–17.5 times higher), mainly due to the distribution of electronic, construction, and logistics industries around Baiyun Lake, which are more influenced by human activities [32]. The levels of Cd, Cu, Pb, Zn, and As in Longyang Lake and Moshui Lake in Wuhan are slightly higher than those in Kaifeng West Lake, mainly due to local industrial emissions, fish farming, and animal feces [33]. The heavy metal content in the sediment of Hulun Lake and Qinghai Lake is mostly lower than that in Kaifeng West Lake, while Chenghai Lake has higher levels of heavy metals other than Zn than Kaifeng West Lake. This is mainly because it is located in an area of atmospheric heavy metal deposition in China, where more heavy metals are released from nonferrous metal smelting. Furthermore, agricultural activities in the region also contribute to heavy metal pollution, which enters the lake through processes such as water and soil erosion [36].

According to the results of the Geo-Accumulation Index and Potential Ecological Risk Index evaluation, Cd contamination is the most severe in the sediment of West Lake in Kaifeng, followed by Cu and Zn. Therefore, this study primarily focuses on the sources of these three heavy metals and does not discuss other elements for now. Based on the comprehensive analysis of Pearson correlation, principal component analysis, cluster analysis, and PMF model results, there is a significant positive correlation between Cd and Cu, as well as Zn, indicating that they can be grouped together.

Previous studies have shown that Cu is commonly used in the metal components of vehicles, and the wearing of materials in car brakes and oil pumps can release Cu into the environment [37,38,39]. Zn primarily originates from traffic emissions, the use of lubricating oils, and gasoline, with tire wear being another major source of Zn [39,40,41,42]. Cd is often found in alloys, galvanized metals, tires, and other materials used in ships and cars, making traffic sources a significant contributor to Cd pollution [39,42,43]. Therefore, in the PMF source apportionment results, Factor 2 represents traffic emissions. Although Factor 1 exhibited the highest contribution (34.9%), primarily derived from As, Ni, and Cr, the risk assessment indicated low risks associated with these elements. Furthermore, the average concentrations of As and Ni did not exceed the soil background values. Therefore, Factor 1 is attributed to natural sources.

On the west bank of West Lake in Kaifeng, there is an artificial beach, and water-based yacht activities are available, resulting in a high density of tourists and many visitors. Consequently, the mechanical wear of yachts, gasoline combustion, and other factors can contribute to heavy metal pollution in the lake sediment. The most severely polluted area is the lake area between Dongjing Road and Zhengkai Road (Figure 2), which is precisely the navigation zone for yachts. Dongjing Road and Zhengkai Road span across Kaifeng West Lake. As important transportation arteries in Kaifeng City, they experience heavy traffic flow and a large volume of goods transportation, leading to pollution from activities such as gasoline combustion, car tire usage, and mechanical wear. These pollutants contribute to the accumulation of heavy metals in the lake sediment. Therefore, traffic factors are a significant cause of heavy metal pollution in Kaifeng West Lake, accounting for 34.3% of the overall pollution. Consequently, regular inspections of scenic facilities should be conducted, and the management and pollution control of the lake’s surrounding environment should be strengthened in order to effectively suppress heavy metal pollution in Kaifeng West Lake and reduce ecological risks to the lake.

## 5. Conclusions

This study reveals that while heavy metal concentrations in the surface water of Kaifeng West Lake are low and comply with relevant standards, sediment analysis indicates significant contamination. Key heavy metals (Cd, Cu, Zn, Cr) in the sediment exhibit elevated levels compared to Chinese tidal soil background values, with Cd displaying the most pronounced enrichment and pollution. Spatially, high concentrations of heavy metals are predominantly concentrated in the central lake area, particularly between Dongjing Road and Zhengkai Road, strongly associated with intensive human activities such as tourism and yacht operations. Assessment using the geo-accumulation index confirmed moderate pollution by Cd and mild pollution by Zn and Cu, while other metals were relatively clean. The Potential Ecological Risk Index further identified Cd as posing a moderate ecological risk to the lake ecosystem, with other metals presenting only slight risks. Source apportionment through multiple statistical methods (correlation, PCA, CA, PMF) consistently indicates that the primary pollutants (Cd, Cu, Zn) share similar origins, predominantly attributed to transportation-related sources including traffic emissions, yacht mechanical wear, and gasoline combustion.

## Figures and Tables

**Table 1:** Revised classification criteria for *E* and RI.

*E*	Ecological Risk Levels of a Single Metal	RI	Ecological Risk Levels of a Single Metal
<30	Low ecological risk	<70	Low ecological risk
30~60	Moderate ecological risk	70~140	Moderate ecological risk
60~120	Considerable ecological risk	140~280	Considerable ecological risk
120~240	High ecological risk	280~560	Very high ecological risk
≥240	Very high ecological risk		

**Table 2:** Comparison of heavy metal content in the surface water of Kaifeng West Lake with Environmental Quality Standards (μg·L^−1^).

Elements (μg·L^−1^)	Cd	Cr	Cu	Ni	Pb	Zn	As
Mean value	0.030	6.180	3.350	4.400	0.010	10.170	0.940
Maximum value	0.090	9.410	6.370	6.390	0.050	21.250	1.760
Minimum value	0.010	3.750	1.520	2.830	0	4.150	0
GB 3838-2002 *	≤5	≤50	≤1000	≤20	≤50	≤2000	≤100

* In GB 3838-2002, the limit for Ni is specified as the specific project standard limit for centralized domestic drinking water surface sources, while the limits for other heavy metals are classified under Class IV standards.

**Table 3:** Statistical analysis of heavy metal content in sediment of Kaifeng West Lake (mg·kg^−1^).

Elements (mg·kg^−1^)	Cd	Cr	Cu	Ni	Pb	Zn	As
Mean value	0.280	99.950	41.400	28.070	20.410	115.230	3.990
Maximum value	0.410	152.500	73.730	39.220	29.140	147.850	5.750
Minimum value	0.140	47.230	22.090	9.260	17.070	68.700	2.030
Standard deviation	0.060	21.790	11.790	7.110	3.000	16.560	1.000
Background value	0.600	66.600	24.100	29.600	21.900	71.100	9.700
Coefficient of variation/%	21.64	21.80	28.48	25.32	14.72	14.37	25.08

**Table 4:** Geo-Accumulation Index of heavy metals in Kaifeng West Lake sediment.

Elements	Cd		Cr		Cu		Ni		Pb		Zn		As	
Sampling Points	*I_geo_*	*I_geo_* Class	*I_geo_*	*I_geo_* Class	*I_geo_*	*I_geo_* Class	*I_geo_*	*I_geo_* Class	*I_geo_*	*I_geo_* Class	*I_geo_*	*I_geo_* Class	*I_geo_*	*I_geo_* Class
1	0.970	1	0.360	1	0.110	1	−0.930	0	−0.700	0	0.060	1	−2.030	0
2	0.580	1	0.650	1	−0.030	0	−0.300	0	−0.810	0	0.030	1	−2.310	0
3	0.910	1	−0.130	0	0.160	1	−0.670	0	−0.710	0	0.200	1	−1.930	0
4	1.150	2	0.070	1	1.000	1	−0.500	0	−0.660	0	0.380	1	−2.270	0
5	1.440	2	0.480	1	0.340	1	−0.340	0	−0.420	0	0.230	1	−1.410	0
6	1.600	2	0.030	1	1.100	2	−0.200	0	−0.200	0	0.370	1	−1.370	0
7	1.030	2	−0.040	0	0.290	1	−0.600	0	−0.690	0	0.050	1	−1.720	0
8	0.800	1	0.130	1	0.07	1	−0.600	0	−0.760	0	0.070	1	−1.580	0
9	1.050	2	−0.020	0	0.100	1	−0.620	0	−0.610	0	0.170	1	−1.670	0
10	0.740	1	−0.250	0	−0.010	0	−0.740	0	−0.620	0	−0.040	0	−1.540	0
11	0.830	1	−0.180	0	−0.130	0	−1.000	0	−0.860	0	0.080	1	−1.800	0
12	0.080	1	−1.040	0	−0.640	0	−2.190	0	−0.750	0	−0.570	0	−2.780	0
13	1.110	2	0.130	1	0.280	1	−0.250	0	−0.080	0	0.390	1	−1.630	0
14	1.310	2	0.210	1	0.600	1	−0.110	0	−0.480	0	0.410	1	−1.390	0
15	0.800	1	−0.070	0	0.360	1	−0.550	0	−0.710	0	0.220	1	−1.780	0
16	1.360	2	0.300	1	0.390	1	−0.100	0	−0.460	0	0.540	1	−1.280	0
17	1.170	2	−0.230	0	−0.150	0	−1.010	0	−0.790	0	0.160	1	−2.350	0
18	1.080	2	0.040	1	0.130	1	−0.730	0	−0.710	0	0.040	1	−1.990	0
19	0.930	1	0.010	1	0.170	1	−0.730	0	−0.690	0	0.190	1	−1.860	0
20	1.080	2	−0.230	0	0.060	1	−0.940	0	−0.620	0	0.300	1	−2.250	0
21	1.390	2	−0.130	0	0.420	1	−0.420	0	−0.520	0	0.160	1	−1.970	0
average *I_geo_*	1.020	2	0.000	0	0.220	1	−0.640	0	−0.610	0	0.160	1	−1.850	0

**Table 5:** Correlation relationships among heavy metals in sediment.

Elements	Cd	Cr	Cu	Ni	Pb	Zn	As
Cd	1						
Cr	0.32	1					
Cu	0.71 **	0.26	1				
Ni	0.67 **	0.69 **	0.65 **	1			
Pb	0.64 **	0.19	0.54 *	0.62 **	1		
Zn	0.75 **	0.42	0.67 **	0.79 **	0.58 **	1	
As	0.58 **	0.36	0.42	0.71 **	0.57 **	0.57 **	1

Note: ** indicates significance at the 0.01 level, while * indicates significance at the 0.05 level.

**Table 6:** Comparison of heavy metal content in sediment of Kaifeng West Lake and other lakes.

Elements (mg·kg^−1^)	Cd	Cr	Cu	Ni	Pb	Zn	As	Reference
West Lake	0.280	99.950	41.400	28.070	20.410	115.230	3.990	This study
Baogong Lake	0.430	65.820	117.600	31.140	81.960	173.990	—	[31]
Longting Lake	0.340	46.010	30.700	24.660	60.290	95.030	—	[31]
Tieta Lake	0.270	42.270	29.150	20.000	55.920	68.330	—	[32]
Baiyun Lake	4.900	143.100	275.900	117.300	89.500	594.400	—	[32]
Longyang Lake	0.580	59.200	55.610	—	33.200	176.940	14.270	[33]
Moshui Lake	0.620	85.530	53.670	—	41.690	266.640	19.260	[33]
Hulun Lake	0.190	37.070	25.360	—	21.840	50.560	21.440	[34]
Qinghai Lake	—	80.070	27.210	11.620	24.840	110.390	—	[35]
Chenghai Lake	0.680	—	56.200	47.600	28.100	109.000	12.100	[36]

Note: “—” indicates no available data.

## Data Availability

The datasets are available from the corresponding author upon reasonable request.
